# Involvement of Nitric Oxide on *Bothropoides insularis* Venom Biological Effects on Murine Macrophages *In Vitro*

**DOI:** 10.1371/journal.pone.0151029

**Published:** 2016-03-14

**Authors:** Ramon R. P. P. B. de Menezes, Clarissa P. Mello, Dânya B. Lima, Louise D. Tessarolo, Tiago Lima Sampaio, Lívia C. F. Paes, Natacha T. Q. Alves, Eudmar M. Assis Junior, Roberto C. P. Lima Junior, Marcos H. Toyama, Alice M. C. Martins

**Affiliations:** 1 Department of Physiology and Pharmacology, School of Medicine, Federal University of Ceará, Fortaleza, Ceará, Brazil; 2 Department of Clinical and Toxicological Analysis, Federal University of Ceará, Fortaleza, Ceará, Brazil; 3 University of Bristol, School of Biochemistry, Bristol, United Kingdom; 4 São Vicente Unit, Paulista Coastal Campus, São Paulo State University (UNESP), São Paulo, Brazil; Universidade Federal do Rio de Janeiro, BRAZIL

## Abstract

*Viperidae* venom has several local and systemic effects, such as pain, edema, inflammation, kidney failure and coagulopathy. Additionally, bothropic venom and its isolated components directly interfere on cellular metabolism, causing alterations such as cell death and proliferation. Inflammatory cells are particularly involved in pathological envenomation mechanisms due to their capacity of releasing many mediators, such as nitric oxide (NO). NO has many effects on cell viability and it is associated to the development of inflammation and tissue damage caused by *Bothrops* and *Bothropoides* venom. *Bothropoides insularis* is a snake found only in Queimada Grande Island, which has markedly toxic venom. Thus, the aim of this work was to evaluate the biological effects of *Bothropoides insularis* venom (BiV) on RAW 264.7 cells and assess NO involvement. The venom was submitted to colorimetric assays to identify the presence of some enzymatic components. We observed that BiV induced H_2_O_2_ production and showed proteolytic and phospholipasic activities. RAW 264.7 murine macrophages were incubated with different concentrations of BiV and then cell viability was assessed by MTT reduction assay after 2, 6, 12 and 24 hours of incubation. A time- and concentration-dependent effect was observed, with a tendency to cell proliferation at lower BiV concentrations and cell death at higher concentrations. The cytotoxic effect was confirmed after lactate dehydrogenase (LDH) measurement in the supernatant from the experimental groups. Flow cytometry analyses revealed that necrosis is the main cell death pathway caused by BiV. Also, BiV induced NO release. The inhibition of both proliferative and cytotoxic effects with L-NAME were demonstrated, indicating that NO is important for these effects. Finally, BiV induced an increase in iNOS expression. Altogether, these results demonstrate that *B*. *insularis* venom have proliferative and cytotoxic effects on macrophages, with necrosis participation. We also suggest that BiV acts by inducing iNOS expression and causing NO release.

## Introduction

Snake venoms consist of 95% proteins, with or without enzymatic activities, such as neurotoxins, cardiotoxins, lecithins, disintegrins, L-amino acid oxidases (svLAAOs) natriuretic peptides, metalloproteases (svMPs), phospholipases A_2_ (svPLA_2_) and phosphodiesterases [1–3]. These proteins are the main responsible agents for the toxic effects observed in animal and human envenomations.

*Bothrops* and *Bothropoides* venoms show a variety of local and systemic effects. Main local effects include intense pain, edema, local hemorrhage, inflammation and tissue necrosis [4], while the most important systemic effects are acute kidney failure, coagulopathy, arterial hypotension, hemodynamic alterations and intravascular hemolysis [5]. Although the severity of these effects is associated with many factors, their qualitative and quantitative composition is essential to determine the type and intensity of their toxicity [6].

These venoms, as well as their proteins, have shown marked cytotoxic effects on many cell types [7,8]. It has been postulated that the study of this cytotoxicity may help to elucidate the pathological mechanisms of toxicity in snakebites [9]. Events such as cell death, proliferation and expression of mediators can interfere with both their function and tissue response to the venom-induced lesion [8,10].

Moreover, it is known that inflammation plays an important role in the development of snake venom toxicity. It is closely associated to the onset of local and systemic toxicity induced by bothropic venom and its fractions, as described by several authors [11–13]. Additionally, some proteins in these venoms have shown effects on cells associated to inflammation, such as macrophages [14–17] and neutrophils [15,18]. However, the mechanisms involved in these effects are not well known, as well as whether these effects may contribute to the clinical outcomes in snakebites.

*Bothropoides insularis* is a native snake from Queimada Grande Island, located in São Paulo, Brazil. Authors suggest that this species was differentiated from *B*. *jararaca*, due to the geographic isolation of the island [19]. Its venom shows marked toxicity, as described by several authors [20,21].

Therefore, the aim of the present work was to study the biological effects induced by *Bothropoides insularis* (BiV) venom on cultured RAW 264.7 cells and evaluate the participation of NO in its action mechanism.

## Material and Methods

### Venom and chemicals

*Bothropoides insularis* venom (BiV) was kindly donated by the Biochemistry Department of Universidade Estadual de São Paulo (UNESP). For all experiments, the venom was diluted in sterile Phosphate-Buffered Saline (PBS), at a pH of 7.4.

Azocasein, o-phenylenediamine, 4-nitro-3-octanoyloxy-benzoic acid (4N3OBA) and peroxidase were purchased from Sigma-Aldrich (St. Louis, USA).

### *In vitro* enzymatic assays

#### Proteolytic assay

In order to evaluate the presence of metalloproteinases in BiV, its proteolytic activity was assessed as described by Rucavado *et al*. [22], at the concentrations used in our biological activity experiments. Briefly, BiV was mixed with azocasein (5 mg/mL) diluted in reaction buffer (containing 25 mM Tris-HCl, 150 mM NaCl, 5 mM CaCl_2_, pH 7.4) in 96-well plates. After 1 hour of incubation at 37°C, the reaction was stopped by the addition of 5% trichloroacetic acid. The plates were centrifuged at 3500 rpm for 5 minutes, and then the supernatants were transferred to new plates. Finally, 0.5 M NaOH was added and the plates were read at 450 nm (Asys UV Expert Plus®, Cambridge, UK). One unit of proteolytic activity was considered as an increase of 0.01 on absorbance [23].

#### Evaluation of hydrogen peroxide production

The presence of L-amino acid oxidase was analyzed by a colorimetric assay [24]. The reaction mixture (50 μg/mL o-phenylenediamine, 50 μg/mL L-leucine and 0.81 U/mL peroxidase, diluted in 0.1 M Tris-HCl buffer, pH 7.4) was mixed with BiV and incubated for 1 hour at 37°C. The reading was performed at 492 nm after addition of H_2_SO_4_. H_2_O_2_ concentrations were calculated by extrapolation from H_2_O_2_ standard curve.

#### PLA2 assay

BiV was mixed with reaction buffer (10 mM Tris-HCl, 10 mM CaCl_2_, 100 mM NaCl, pH 8.0) containing 4-nitro-3-octanoyloxy-benzoic acid (2 mM 4N3OBA,). Absorbance at 425 nm was read after 20 minutes of incubation at 37 ºC [25].

### Cell maintenance and experimental design

RAW 264.7 cells, a murine macrophage cell line obtained from the peritoneal cavity, were purchased from Rio de Janeiro Cell Bank, and cultured in sterile plastic flasks, using Dulbecco’s Modified Eagle Medium (DMEM) supplemented with antibiotics and fetal bovine serum (10% FBS) in a CO_2_ incubator (Tecnal, São Paulo, Brazil) at 37°C until confluence was reached. For the experiments, confluent cultures were washed with PBS, dislocated with trypsin/EDTA, centrifuged (4000 rpm, 5 minutes) and quantified in a Neubauer Chamber, using trypan blue as a vital stain. Finally, the cells were plated at 1 × 10^5^ cells/mL in 24 or 96-well flat bottom sterile plates. Sterile PBS was used as negative control. After 24 hours of incubation, BiV was added at several concentrations to perform the methods described below.

### Viability assays

#### MTT reduction assay

Cell viability was assessed as described by Mosmann [26], using MTT (3-(4,5-dimethylthiazol-2-yl)-2,5-diphenyltetrazolium bromide), a yellow tetrazole dye, which is reduced to formazan by cytoplasmic and mitochondrial reductases present in viable cells. For this purpose, 96-well plates containing RAW 264.7 cells treated with BiV (200, 100, 50, 25, 12.5 and 6.25 μg/mL) for 2, 6, 12 or 24 hours were treated with MTT 2.5 mg/mL (Sigma-Aldrich, St. Louis, USA) for 4 hours in the dark. The resulting formazan was solubilized by adding 10% sodium dodecyl sulphate (SDS). After 17 hours, the plates were read at 570 nm.

#### Trypan blue method

The cells were harvested in 24-well plates and treated with BiV for 24 hours. The experimental groups were dislocated, centrifuged and the pellet was diluted in trypan blue 0.1%. The number of viable cells was determined by counting in Neubauer Chamber.

#### Lactate dehydrogenase (LDH) release measurement

LDH is a cytoplasmic enzyme, which is released when plasmatic membrane damage occurs [27]. Thus, in order to investigate cell lysis induced by *B*. *insularis* venom, the supernatant of the MTT assay experimental groups was removed to determine LDH activity using a colorimetric kit (Roche, Mannheim, Germany). The method is based on the NAD^+^/NADH+H conversion induced by the enzyme. NADH+H is used do convert a tetrazolium salt into a red product, read at 490 nm.

### Nitrate/nitrite measurement

Supernatants from cells treated with BiV for 24 hours were collected to determine NO production, through measurement of nitrite/nitrate levels, using a commercial kit (Roche, Mannheim, Germany). Samples were treated with nitrate reductase (5.7 U/mL) for 30 minutes and then submitted to Griess reaction [28]. Additionally, the cells were treated with 25 μM L-NAME (Nω-Nitro-L-arginine methyl ester, Sigma-Aldrich) for 2 hours before the treatment with BiV [29], to evaluate the efficacy of NO production blockage by L-NAME.

### Flow cytometry

After 24 hours of treatment, RAW 264.7 macrophages were submitted to flow cytometry analysis, to determinate cell death mechanisms involved in BiV cytotoxic effect. The cells were dislocated, centrifuged and washed twice with binding buffer (10mM Hepes, 140 mM NaCl, 2.5 mM CaCl_2_, pH 7.4). Treated and untreated cells were labeled with annexin V-PE and 7-aminoactinomycin D (7-AAD) for 15 minutes in the dark. Finally, the experimental groups were analyzed in FACSCalibur flow cytometry device (BD Biosciences, New Jersey, USA) using the CellQuest Pro® software.

### *In vitro* blockage of NO production

To determine whether NO was responsible for BiV biological effects, RAW 263.7 cells were treated with 25 μM L-NAME for 2 hours, treated with BiV and submitted to MTT assay after 24 hours. These results were compared to the effect of BiV without the pre-treatment with L-NAME.

### Inducible Nitric Oxide Synthase (iNOS) expression evaluation

iNOS expression was assessed by western blotting, as performed by Chae *et al*. [30], with modifications. Briefly, cells were collected and washed twice with cold PBS. Pellets were resuspended and lyzed with RIPA buffer (Tris 50mM; NaCl 150mM; EDTA 1mM; Triton X-100 1%; PMSF 1mM) and centrifuged. We measured total protein in the supernatant and added the volume, which had 20 μg of wild protein with 1:1 sample buffer (Glycerol; Tris; β-mercaptoethanol; Bromophenol Blue; SDS). After that, the mixture was heated in a water bath up to 100°C for 5 minutes and separated in a polyacrylamide SDS-page gel (Bio-Rad, Hercules, CA, USA). Separated proteins were transferred to nitrocellulose membranes, which were incubated with 5% non-fat milk in TBS-T overnight to block the nonspecific binding site. On the next day, the membranes were washed thrice and incubated with primary antibody for 2 hours (1:800 5% non-fat milk TBS-T; NOS2 Antibody M-19 by Santa Cruz Biotechnology®). After that, the membranes were again washed thrice and incubated with secondary antibody for 1 hour (1:1000 5% non-fat milk TBS-T; Anti-Rabbit Antibody by Santa Cruz Biotechnology®), again washed thrice and incubated with the Western Blotting Luminol Reagent (Santa Cruz Biotechnology®), following the manufacturer's recommendations, and finally placed in a radiography frame together with a radiographic film for mammography (FUJI AD-M) for 5 minutes, and the films were developed in an automatic processor and the images were analyzed using ImageJ® program.

### Statistical analysis

All experiments were performed in triplicate (n = 3) and the data were expressed as mean ± Standard Error Mean (SEM). ANOVA was used for comparison of the results with Dunnett’s post-test, with significance being set at p<0.05. The analyses were carried out with GraphPad Prism® software, version 5.0 and Microsoft Excel® 2010.

## Results

**Fig 1:**
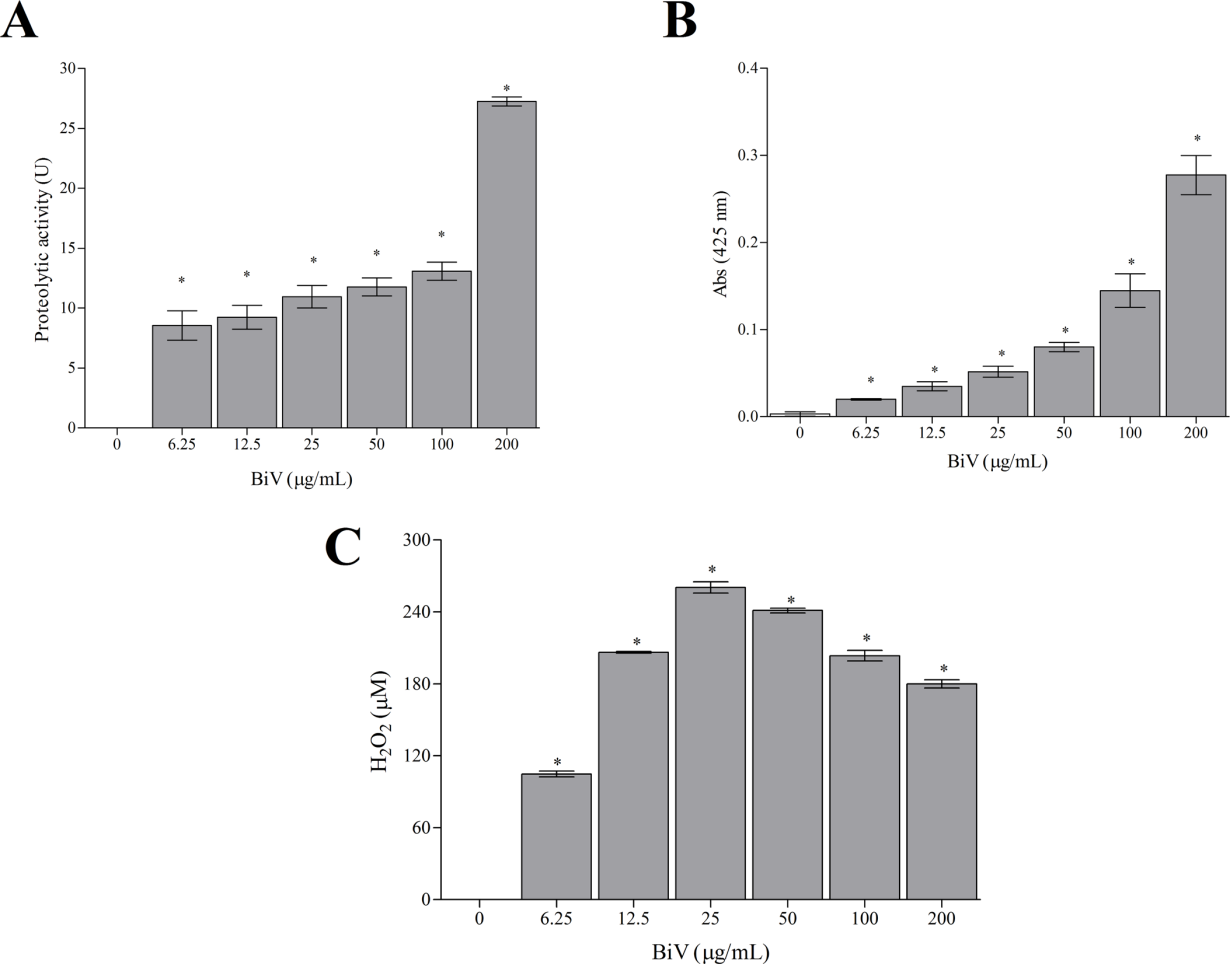
Enzymatic activities exhibited by BiV. [A] Proteolytic activity, assessed by azocasein cleavage method; [B] phospholipasic assay, determined using 4N3OBA as the lipidic substrate; and [C] H_2_O_2_ production induced by BiV, suggestive of the presence of LAAO. *p<0.05 vs control group (absence of venom).

In the present study, the presence of important components of BiV was verified by enzymatic assays, which demonstrated that *B*. *insularis* venom has proteolytic and phospholipasic activities, and results in H_2_O_2_ production. These findings suggest the presence of metalloproteases (svMPs), PLA_2_ and LAAO in BiV, respectively (Fig 1). It is important to highlight that these assays were performed at the same concentrations used in the other experiments, aiming to identify possible fractions responsible for BiV biological effects.

**Fig 2:**
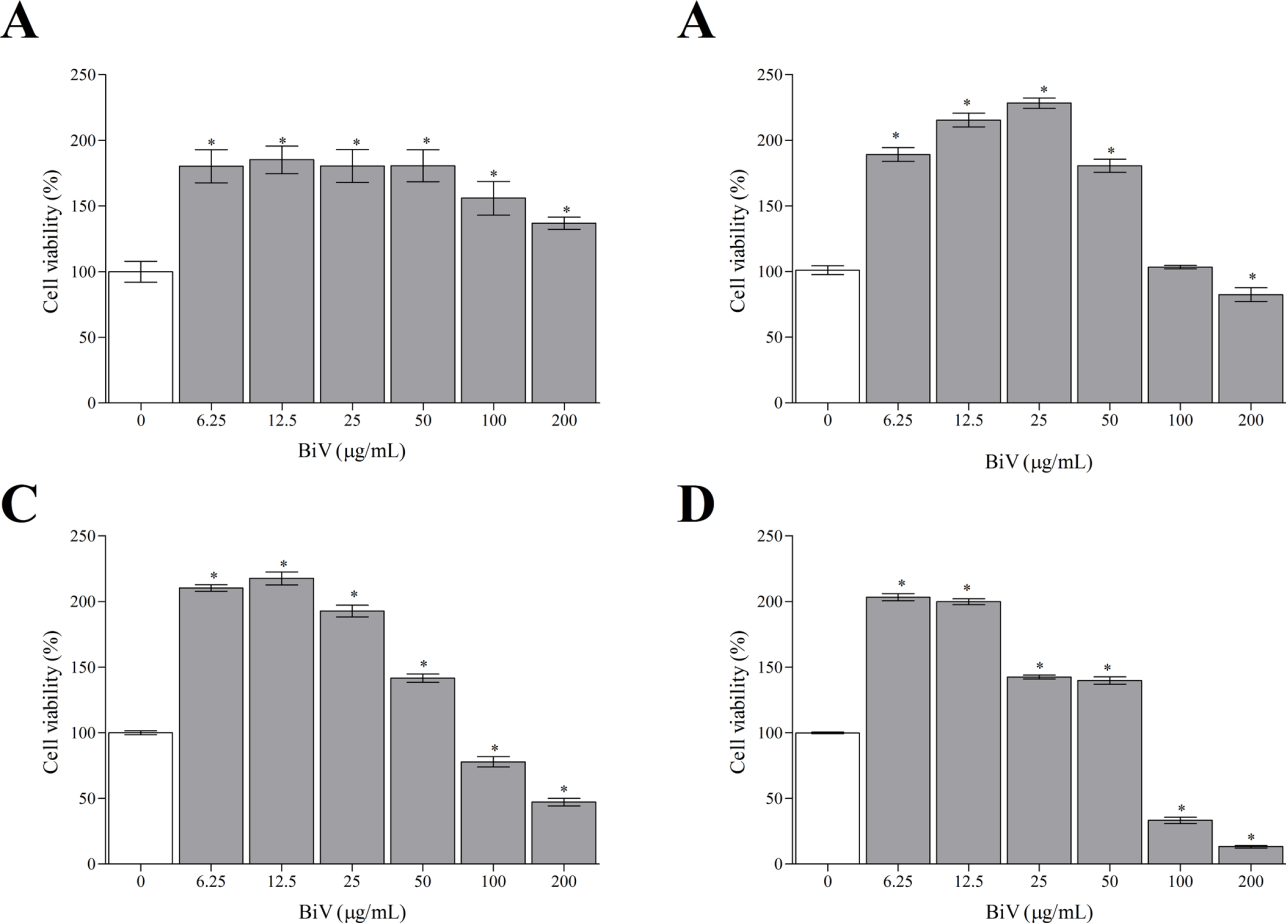
Time- and concentration-dependent biological effect caused by BiV on RAW 264.7 cells. Cell viability was determined after [A] 2 hours, [B] 6 hours, [C] 12 hours and [D] 24 hours of incubation with BiV, using MTT reduction assay. *p<0.05 vs. control group.

**Fig 3:**
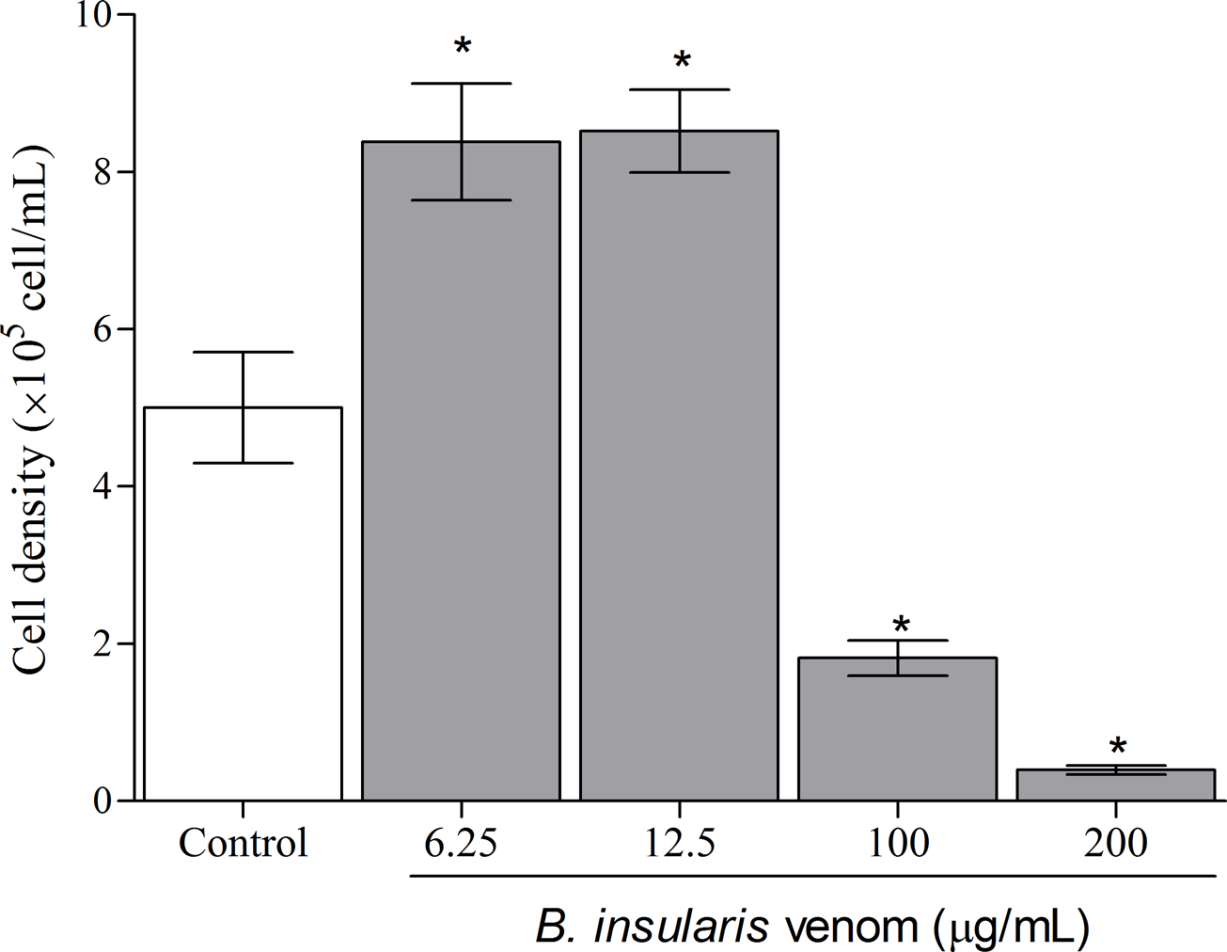
Cell number in machophages treated with BiV. The cell density was determined by counting in Neubauer chamber. *p<0.05 vs. negative control group.

**Fig 4:**
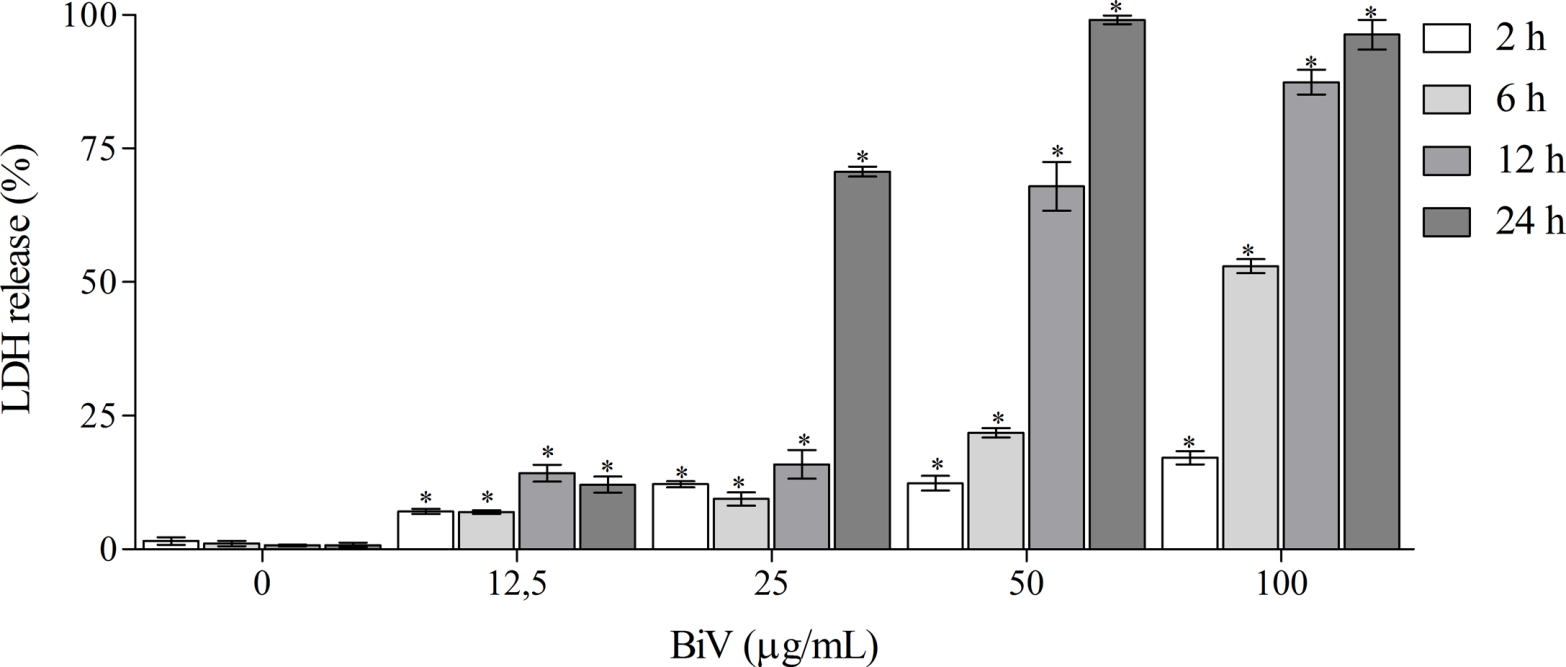
LDH release in macrophages treated with BiV. The percentage of enzymatic release was calculated using a group treated with 1% Triton X-100 as total cell lysis control (data not shown). *p<0.05 vs. negative control group.

In the present study, macrophages exposed to BiV showed a concentration- and time-dependent effect when submitted to MTT assay. After 2 hours (Fig 2A), all experimental groups treated with the venom showed an increase in cell viability, suggesting a possible proliferative effect. The groups exposed for 6, 12 and 24 hours (Fig 2B, 2C and 2D) showed dual effects, with cell death at high concentrations and increased cell viability at lower concentrations of BiV. It was also observed an increase on the number of cells treated with BiV at lower concentrations, when analyzed by counting with trypan blue (Fig 3). This cytotoxic effect was confirmed by the determination of LDH activity in the supernatant of experimental groups (Fig 4).

**Fig 5:**
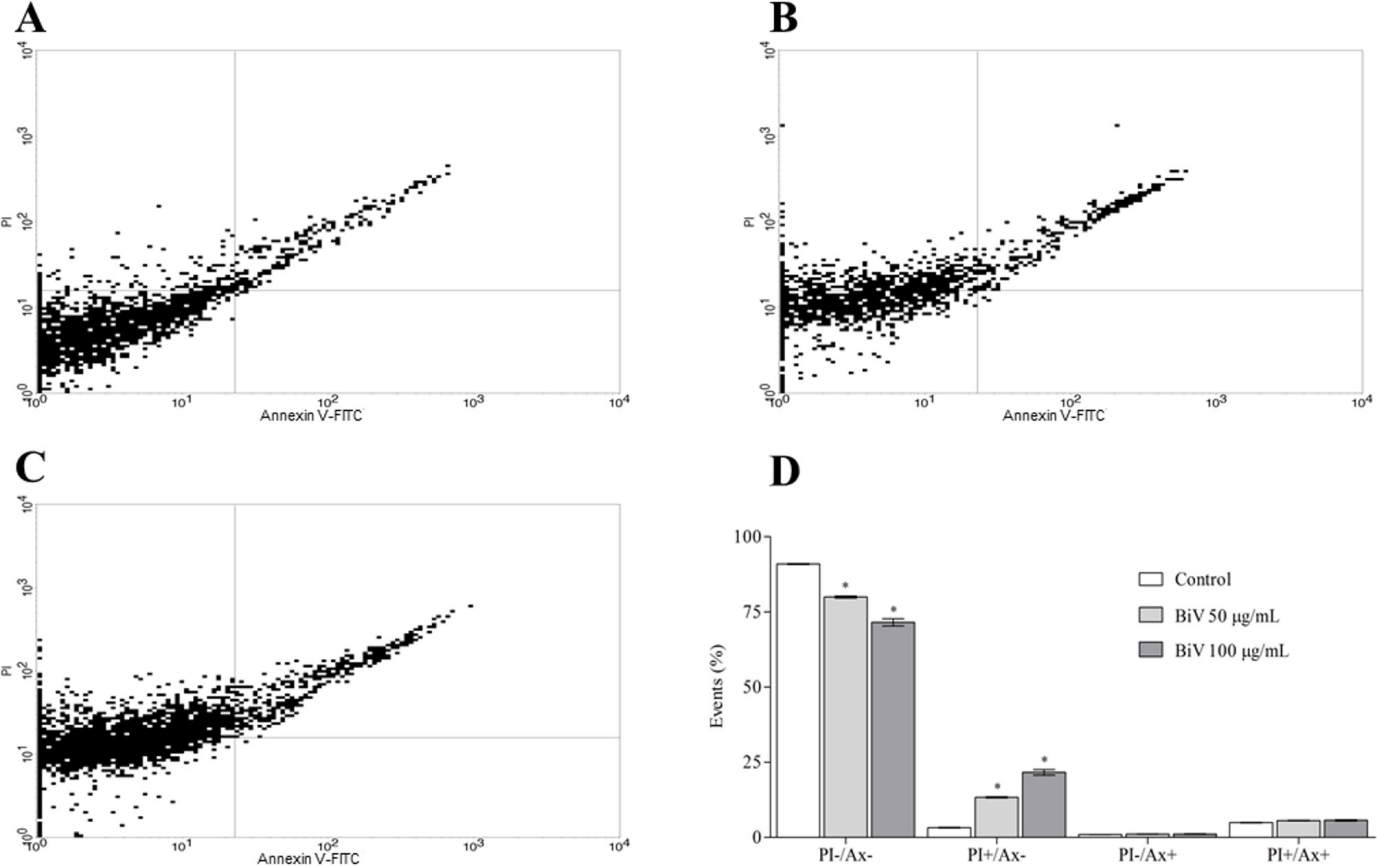
Necrotic potential of BiV on murine macrophages *in vitro*. [A-C] Dot plot diagrams representing respectively control and groups treated with BiV at 50 μg/mL and 100 μg/mL. [D] Increase in the percentage of PI-labeled cells, suggestive of cell death by necrosis. *p<0.05 vs. control group.

In order to investigate the cell death mechanism induced by BiV, treated and untreated cells were collected, washed and labeled with PI and Annexin V-FITC. As result, it was observed that BiV induced an increase in PI^+^ cells (Fig 5). These results indicate that BiV induced necrosis in the analyzed cells.

In the present study, we observed that *B*. *insularis* venom causes not only cell death of macrophages, but also have a potential proliferative effect. It is known that inflammatory cells, such as macrophages, can respond to damaging effects through cell activation and production of inflammatory mediators. These mediators are partially responsible by both local and systemic alterations observed in *Viperidae* envenomation.

**Fig 6:**
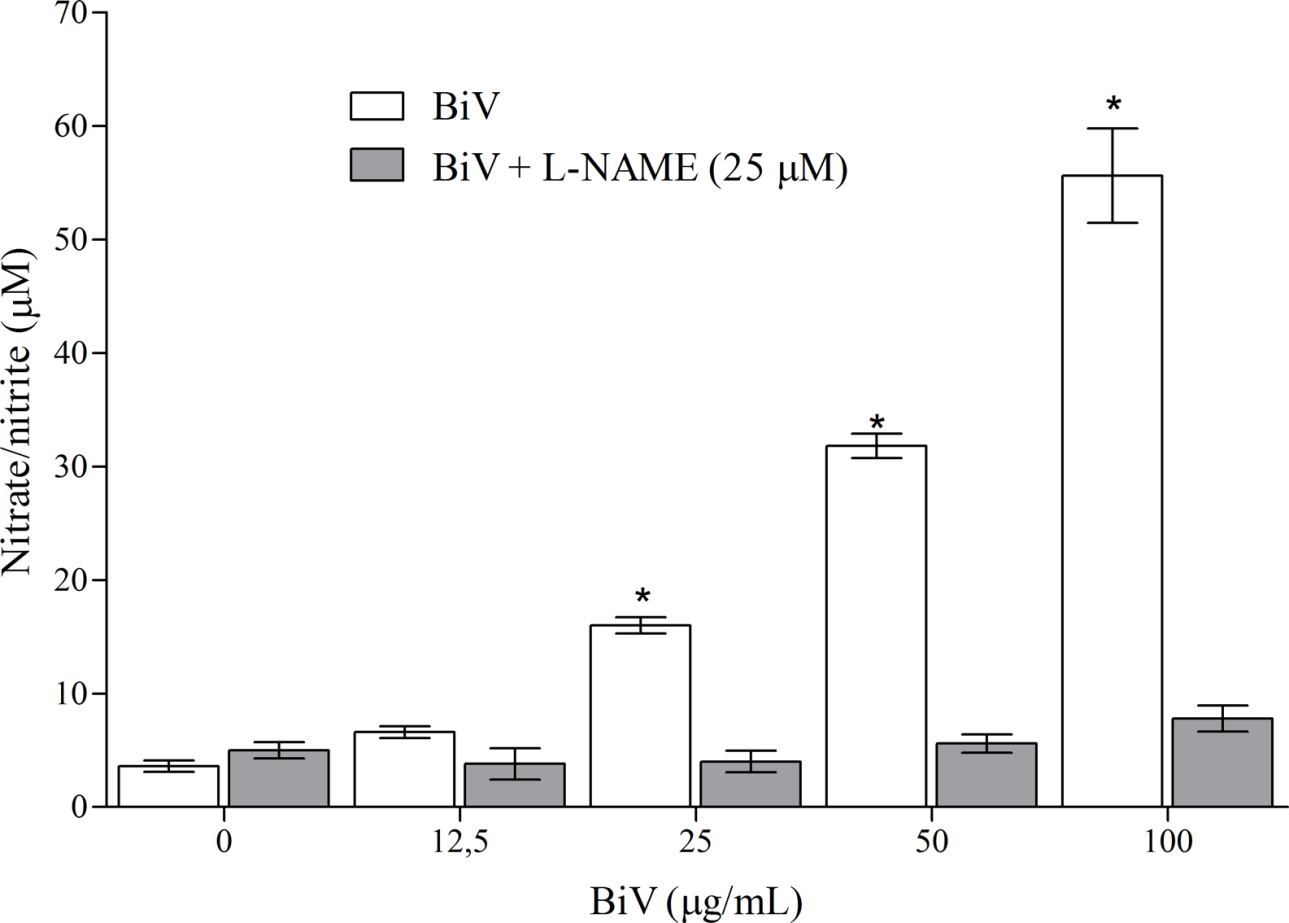
Analyses of nitrate/nitrite concentrations in macrophages after treatment with BiV with and without L-NAME. The analysis was performed using Griess method with modifications. *p<0.05 vs. control group.

Consequently, aiming to identify the participation of nitric oxide on BiV biological effects, the supernatants from experimental groups treated for 24 hours were collected to determine nitrate/nitrite concentrations as markers of NO production. As shown in Fig 6, increases in nitrate/nitrite levels in the groups treated with BiV (25, 50 and 100 μg/mL) were observed. Also, NO release was successfully blocked by the addition of L-NAME (25 μM), suggesting the importance of iNOS pathway in its production. L-NAME is a nonselective inhibitor of NO synthase, which blocks NO production. Therefore, we suggest that NO is released by macrophages after incubation with BiV by activation of L-arginine-nitric oxide-cGMP pathway.

**Fig 7:**
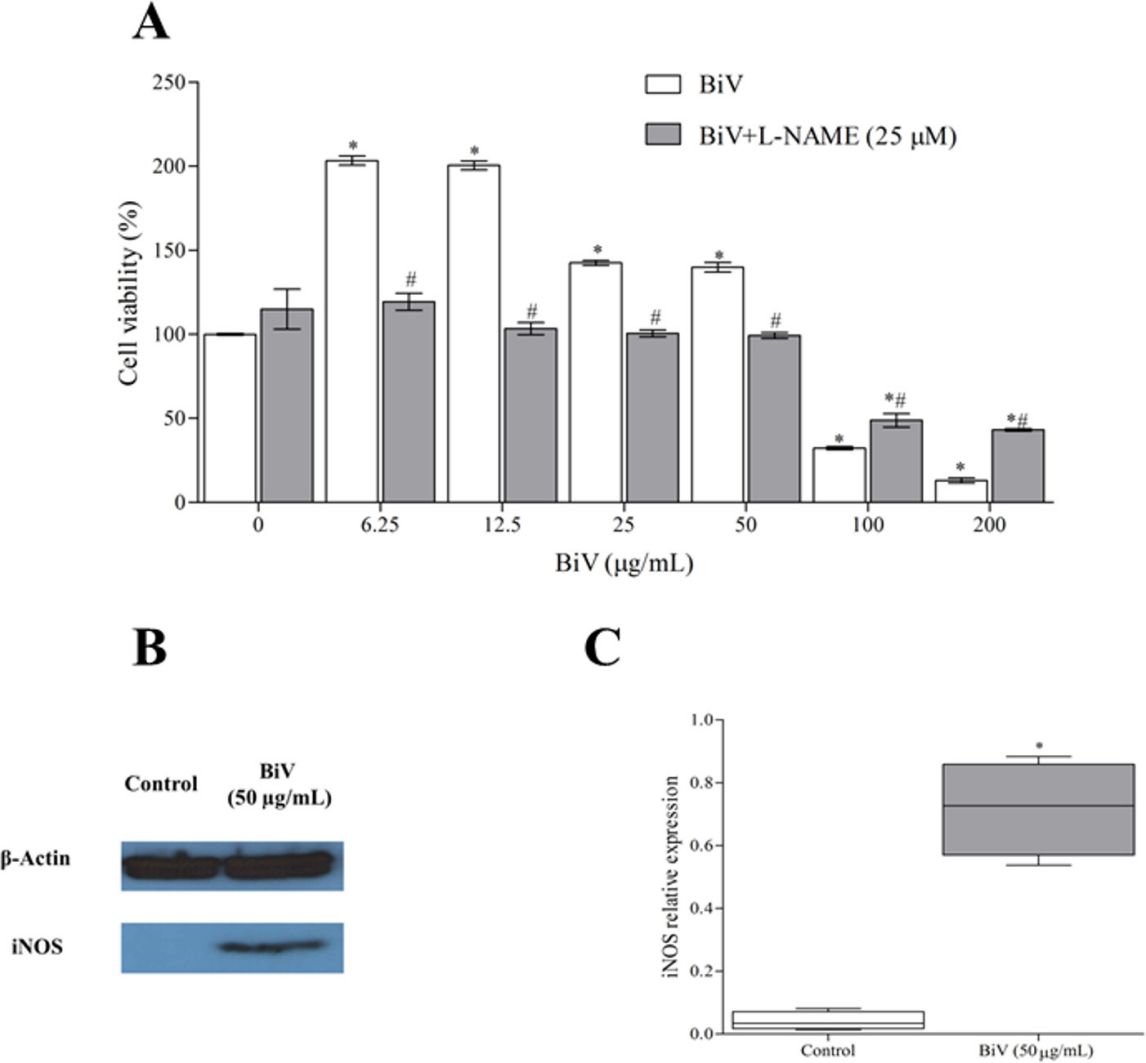
Involvement of NO in BiV biological effects. [A] L-NAME (25 μM) inhibited both cytotoxic and proliferative effects of BiV, assessed by MTT method. [B] Western blotting diagrams representing the expression of β-actin and iNOS in RAW 264.7 cells in control group and treated with BiV (50 μg/mL). [C] Relative expression of iNOS, correlated with β-actin expression. *p<0.05 vs. control group.

To investigate how NO influences BiV effect on RAW 264.7 cell viability, we performed MTT assays in cells treated with BiV for 24 hours in the presence of L-NAME (25 μM). L-NAME was able to reduce the cytotoxic and proliferative effects of BiV (Fig 7A), suggesting that NO is associated to both pathways. Also, it was demonstrated that BiV induced an increase in iNOS expression (Fig 7B and 7C).

## Discussion

Pathological events associated with snakebites are complex and have several variables, such as the bite site, amount of venom injected and, specially, venom composition. Therefore, research groups worldwide have sought to elucidate the biological mechanisms involved in these effects, as well the main proteins responsible for the toxicity. This strategy may contribute to the development of effective treatments and adequate clinical procedures. *B*. *insularis* venom is considered an important source of bioactive and/or toxic proteins, being valuable as a pharmacological tool.

Although many studies have demonstrated inflammatory influence on the clinical outcomes in snakebites, the direct effects of *Viperidae* venoms on cells associated with inflammatory response are not well known, as well as the role of these effects on the consequences of envenomation.

The enzymatic activities observed in this work have been corroborated by other authors. Lira *et al*. [31] demonstrated that BiV has important proteolytic and phospholipasic activities. Also, many authors have isolated and characterized these proteins in other bothropic venoms, such as svMPs isolated from *B*. *alternatus* and *B*. *pauloensis* venoms [32,33]; svPLA_2_, identified in *B*. *pauloensis* and *B*. *leucurus* [34,35]; and LAAOs, which has been identified in several venoms, such as *B*. *jararacussu*, *B*. *marajoensis* and *B*. *pirajai* [36–38].

Valente *et al*. [39] found out that almost 1/4 of the total proteins in BiV correspond to svMPs, which may explain its high myotoxicity and cytotoxicity [40]. Different svPLA_2_ isoforms and a svLAAO have also been identified.

In the present paper, we demonstrate that BiV causes both cell death and proliferation on macrophages, depending of concentration and exposure time. These results are supported by the MTT assay, which verifies the metabolic ability of cells, and by trypan blue method, which assess membrane integrity and number of viable cells. Different *Bothrops* and *Bothropoides* venoms have influence on cell viability. In a previous study, our group showed a similar effect using *Bothropoides lutzi* venom [41]. Recently, we also demonstrated that BiV has a marked cytotoxic effect on kidney cells, as demonstrated by Mello et al. [42]. In this work, BiV showed high toxicity at 200 μg/mL, which is also the concentration with the highest proteolytic and phospholipasic activities.

This corroborates other studies, as bothropic venom cytotoxicity is commonly associated with svMPs and svPLA_2_. For instance, svPLA_2_ isolated from *B*. *alternatus* and *B*. *jararacussu* show cytotoxic effect on muscle cells [43,44]. svMPs with cytotoxic effect include the ones isolated from *B*. *asper* [45,46]. The effects observed in *Bothrops* and *Bothropoides* venoms are probably due to a synergic and/or additive effect between these and other fractions with cytotoxic effect [43]. An important aspect of *B*. *insularis* venom is the presence of an Asp49 PLA_2_, which presents high enzymatic activity when compared to svPLA2 present in other snake venoms, as such *B*. *jararaca* and *B*. *jararacussu* [47]. This might be related to its high toxicity.

Cyototoxicity was also confirmed by LDH measurement, which is an important marker of cell lysis, both in clinical and experimental samples. It is released specially after loss of membrane integrity, a characteristic of cell death by necrosis. *B*. *atrox* venom causes an increase in LDH release in mice [48]; svMP from *B*. *neuwiedi* causes LDH release in endothelial cells [49].

Mello *et al*. (2014) demonstrated That BiV induced necrosis and late apoptosis in kidney tubular cells. Additionally, svPLA_2_ from *B*. *leucurus* acts mainly by necrosis, with Ca^++^ involvement and mitochondrial depolarization [7]. Necrosis is also induced by *B*. *pauloensis*, *B*. *diporus* and *B*. *pirajai* venoms against tumor cells [50]. Necrosis is a cell death pathway that involves release of intracellular content and inflammatory response. Therefore, direct cytotoxicity might be related to local or systemic effects observed in envenomation, such as platelet activation and hemorrhage observed in svMPs from *B*. *leucurus* [51].

Local and systemic toxicity of animal venoms are dependent of the concomitant effect of its several bioactive proteins. For instance, it was demonstrated that blood flow maintaining is necessary to the establishment of muscular damage induced by svMPs [5]. This might be facilitated by vasodilatation promoted through NO production in endothelial cells and macrophages stimulated by svLAAOs and svPLA_2_ [33].

Cytotoxicity can also be induced by svLAAOs, as previously reported [38]. Important functional alterations are caused by svLAAOs, such as DNA damage [52], mitochondrial dysfunction [53] and apoptosis [54].

We decided to investigate the role of nitric oxide on the biological effects found in this work. NO is an important mediator of cell viability and proliferation. The proliferative effect of NO on endothelial cells has been demonstrated [55,56]. Moreover, NO is associated with the cytotoxic mechanism of some drugs [57] and hepatic lesions [58]. In macrophages, NO is produced by activated macrophages as a cytotoxic molecule, aiming at the neutralization of microorganisms [59].

Inflammation induced by bothropic venoms has been associated with NO in several cases. Jararhagin, a svMP isolated from *B*. *jararaca* venom, causes endothelial dysfunction, NO and prostacyclin production, leukocytosis and hemorrhage [60]. It is also important for the development of edema and inflammatory infiltrate in myonecrosis induced by *B*. *asper* venom [61].

However, it is still unclear whether NO influences the tissue lesion found after bothropic snakebite. Chaves *et al*. (61) demonstrated that NO production blockage did not change the muscular lesion induced by *B*. *asper* venom. In this way, it seems that NO is involved in hemorrhage and recruitment of inflammatory cells after lesion, but not directly over tissue lesion.

Beside these results, in vivo blockage of NO production may still be important on management of envenomation, since this mediator is related to establishment of inflammation microenvironment. High tissue NO levels and its metabolites, as such as peroxynitrite, contributes to pathological damage observed in inflammatory site [30]. In contrast, low levels of NO exert protective effect by modulating the immune system [62]. NO management can also be useful in the control of nephrotoxicity induced be snake venoms. It has been suggested that enhance on machophage iNOS expression in kidney after injury aggravates the lesion [63].

Altogether, these results demonstrate the cytotoxic and proliferative effects of BiV on murine macrophages through a necrotic pathway. This result seems to be related mainly to the proteolytic and phospholipasic activities and NO is partially responsible for these effects. We also observed that the venom causes iNOS expression induction.
